# Hepatoprotective effects of Spirulina maxima in patients with non-alcoholic fatty liver disease: a case series

**DOI:** 10.1186/1752-1947-4-103

**Published:** 2010-04-07

**Authors:** Aldo Ferreira-Hermosillo, Patricia V Torres-Duran, Marco A Juarez-Oropeza

**Affiliations:** 1 Department of Biochemistry, School of Medicine, National Autonomous University of Mexico (UNAM), Mexico, 04510, Mexico

## Abstract

**Introduction:**

Non-alcoholic fatty liver diseases range from simple steatosis to non-alcoholic steatohepatitis. The "two hits" hypothesis is widely accepted for its pathogenesis: the first hit is an increased fat flux to the liver, which predisposes our patient to a second hit where increasing free fatty acid oxidation into the mitochondria leads to oxidative stress, lipoperoxidation and a chain reaction with increased ROS. Clinical indications include abdominal cramps, meteorism and fatigue. Most patients, however, are asymptomatic, and diagnosis is based on aminotransferase elevation and ultrasonography (or "brilliant liver"). Spirulina maxima has been experimentally proven to possess *in vivo *and *in vitro *hepatoprotective properties by maintaining the liver lipid profile. This case report evaluates the hepatoprotective effects of orally supplied Spirulina maxima.

**Case presentation:**

Three Hispanic Mexican patients (a 43-year-old man, a 77-year-old man and a 44-year-old woman) underwent ultrasonography and were treated with 4.5 g/day of Spirulina maxima for three months. Their blood samples before and after the treatment determined triacylglycerols, total cholesterol, high-density lipoprotein cholesterol, alanine aminotransferase and low-density lipoprotein cholesterol levels. The results were assessed using ultrasound.

**Conclusion:**

Treatment had therapeutic effects as evidenced by ultrasonography and the aminotransferase data. Hypolipidemic effects were also shown. We conclude that Spirulina maxima may be considered an alternative treatment for patients with non-alcoholic fatty liver diseases and dyslipidemic disorder.

## Introduction

According to the National Institute of Statistics, Geography and Information (INEGI), cirrhosis is the fourth leading cause of death among the general population in Mexico [1]. Its pathology is associated with metabolic syndrome, and insulin resistance is a common pathogenic mechanism. Currently, the known pathologies related to this physiopathological mechanism include non-alcoholic fatty liver disease (NAFLD), which ranges from simple steatosis to non-alcoholic steatohepatitis (NASH) [2]. Each disease has particular histopathological characteristics but these commonly include vesicular steatosis, cellular ballooning, Mallory bodies, diffuse inflammation and pericellular fibrosis [3]. Concerning its pathogenesis, the most accepted theory is the "two hits" theory in which the liver undergoes a first hit through an increased fatty acid flux to the mitochondria. The increase in β-oxidation promotes more adenosine triphosphate (ATP) consumption. This leads to cellular damage, an increase in reactive oxygen species (ROS) [4] and the activation of the immune system. It concludes with the activation of other cells (as fibroblasts) with deposits of collagen types I and IV (which initiate fibrosis). This is known as the second hit [5]. Certain cytokines such as tumor necrosis factor-alpha (TNF-α), transforming growth factor-beta (TGF-β), interleukin-8 (IL-8) and IL-10 also increase and become involved in inflammatory processes and the development of fibrosis [6].

The clinical symptoms of patients include abdominal cramps, meteorism and fatigue. Most patients are asymptomatic. Diagnosis is based on the elevation of aminotransferase levels and unspecific changes seen on an ultrasonography (brilliant liver) and by using MRI [7]. The disease can progress slowly to cirrhosis with a clinical manifestation of portal hypertension. Exclusion criteria include alcohol consumption of more than 30 g per day, serum antibodies to hepatitis B, C or D, evidence of autoimmune diseases and hemochromatosis. Biopsy remains the gold standard for diagnosing NASH with lobular hepatitis, cellular ballooning, periportal infiltrate, and finally fibrosis [8].

Several pharmacological, non-pharmacological and alternative therapy strategies have been investigated. Current treatments include weight loss management and the reversal of the components metabolic syndrome. Drug therapy includes insulin sensitizer therapy, lipid-lowering drugs (which decrease very low-density lipoproteins [VLDL], thus reducing lipid mobilization), ursodeoxycholic acid (which mobilizes hepatotoxic bile acid from their pool), betaine (which increases hepatic S-adenosylmethionine) and antioxidants (vitamin E and N-acetylcysteine). However, no pharmacological treatment to date has been shown to be effective against NAFLD [9].

*Spirulina maxima *is a cyanobacterium that has been used as a food supplement because of its high content of proteins with essential amino acids, carotenoids, B-vitamin complex, minerals and γ-linolenic, ω-3 and ω-6 fatty acids [10]. In previous studies with rats using an intra-peritoneal dose of carbon tetrachloride (CCl_4_) as a hepatotoxin, we found that a purified commercial diet supplemented with 5% Spirulina maxima decreases serum aspartate aminotransferase, liver triacylglycerols (TAG) and total cholesterol (TC). This same pattern was observed in liver free fatty acids (with an important decrease in unsaturated fatty acids) and thiobarbituric acid reactive substances (TBARS, which are indicators for lipoperoxidation) [11]. These results demonstrated that Spirulina has hepatoprotective properties which maintain the liver lipid profile. However, there are currently only a few studies which have evaluated these effects in humans.

## Case presentation

Three Hispanic Mexican patients, aged between 40 and 60 years, were diagnosed with NAFLD by ultrasonography. They were instructed to take 4.5 g/day of Spirulina maxima in tablet form (0.5 g each). An evaluation period of three months was set in order to observe changes in their lipid levels before and after the treatment. During this time all of them were asked to maintain their dietary habits and lifestyles in order to minimize specific effects on their lipid metabolism. At the beginning of the study and then once every month, their blood samples were taken after 12 hours of fasting and 15 minutes of rest. Blood collection tubes were used in order to observe their initial and final levels of TAG, TC, HDL-cholesterol (HDL-C), as well as weekly alanine aminotransferase (ALT) levels to assess the hepatoprotective effect of the treatment.

The protocol and the aim of the study were fully explained to the subjects, who gave their written consent. The research was carried out in accordance with the Declaration of Helsinki and was approved by the Ethics Committee of the School of Medicine, National Autonomous University of Mexico, Mexico City.

Blood samples for plasma analyses were drawn into 100 USP units of sodium heparin (freeze-dried, sterile glass tubes, BD Vacutainer). The TC and TAG concentrations of our patients were analyzed using standard enzymatic procedures (Jas Diagnostics, Inc, Mexico) with a spectrophotometer (Genesys 10 UV, Thermo Electron Corporation, USA). The HDL-C concentration of each blood sample was measured after the precipitation of apo B-containing lipoproteins with the precipitation of the reactive agent (Boehringer Mannheim, Mexico). The LDL-C concentration was determined using the Friedewald equation, while ALT was measured using an enzymatic kit (Sigma Diagnostics, USA) by spectrophotometry methods.

## Case 1

Our first patient was a 43-year-old Hispanic Mexican man with a carbohydrate-based diet. He had smoked one or two cigarettes per day from 18 to 40 years of age. He had been consuming almost 250 mL of red wine per day since he was 20 years old. He also occasionally consumed one bottle of beer. Five years prior to the study, he was diagnosed with hypercholesterolemia and hypothyroidism for which he remained under treatment with levothyroxine 100 μg daily. He was also diagnosed with chronic renal failure eight months prior to the study. His nephrologists did not suggest any specific medical treatment for this condition.

He was diagnosed with NAFLD after he presented with asthenia, fatigue, nausea without vomiting and hypochondrial pain. He was noted to have both elevated cholesterol and TAG levels. An ultrasound examination revealed a "brilliant liver", which is congruent with a diagnosis of fatty liver disease.

His physical examination revealed the following: 1.72 m height, 77 kg weight, body mass index (BMI) of 26 kg/m^2^, blood pressure of 120/90 mmHg, respiratory rate of 21 breaths per minute and a body temperature of 36.5°C. His head and neck were without alterations. His heart had normal sounds and adequate rhythm and frequency. His pulmonary area had adequate ventilation. An exploration of his abdomen showed no clinical signs of significance and his hip-to-waist ratio was 0.75. His genitalia also had no alteration. His musculoskeletal system was found to be normal.

He was then started on a treatment of a low-caloric diet, daily exercise (30-minute walk) and ingestion of Spirulina maxima. Adverse side-effects included flatulence, meteorism and abdominal pain of moderate intensity without irradiation.

## Case 2

Our second patient was a 77-year-old Hispanic Mexican man with a carbohydrate- and fat-based diet. He had smoked two to three cigarettes per day from 17 to 50 years of age. He drank a bottle of beer every weekend. He had a history of penicillin allergy. He had arterial systemic hypertension which evolved over six years and was undergoing treatment with angiotensin-converting enzyme inhibitors at low doses. He suffered an accidental firearm shot when 11 years old, and had a surgical extraction to remove the bullet from his left eye. He had an appendectomy 55 years previously and an abdominal resection for his prostatic hyperplasia 35 years previously.

He was asymptomatic for NAFLD, but an elevated ALT level was detected during a check. An ultrasound examination revealed a "brilliant liver", which corresponds to fatty liver disease.

His physical examination revealed the following: 1.60 m height, 75 kg weight, BMI of 29.9 kg/m^2^, blood pressure of 130/80 mmHg, respiratory rate of 20 breaths per minute and a body temperature of 36.5°C. His head and neck had no alterations. Cardiopulmonary and abdominal examinations of our patient showed normal results, with a hip-to-waist ratio of 0.90. His musculoskeletal system was also normal.

He noted an improvement in his general status during the treatment with Spirulina. No adverse side-effects were shown.

## Case 3

Our third patient was a 44-year-old Hispanic Mexican woman with a carbohydrate-based diet. She led a sedentary lifestyle and had been obese since childhood. She had no history of alcohol or tobacco use. She had a history of allergy to dicloxacillin and ketorolac. In 1998, she was diagnosed with thyroid cancer and secondary hypothyroidism. A thyroidectomy was carried out to remove the tumour. She had continued under treatment with levothyroxine 100 μg three times a week and 150 mg two days per week. She had also been diagnosed with ovarian cancer in 2004, which was followed by surgical treatment. Arterial hypertension was detected in 2004, and she continued under treatment with a β-blocker (metoprolol) 100 mg twice a day.

In June 2006, she experienced a pain in her right hypochondrium and lumbar zone. The pain was constant, oppressive and of moderate intensity (Assessment of Visual Analog AVA 5/10). She had no irradiations, jaundice, dark urine, fatigue, anorexia, xerosis, dry eye or fever. A viral panel for hepatitis A, B and C was negative. She had no elevated bilirubin, but slight increases in her ALT and TC levels were observed. A liver ultrasonography was performed, which detected "brilliant liver". A biopsy confirmed a diagnosis of NAFLD.

Her physical examination revealed the following: 1.59 m height, 76.8 kg weight, BMI of 30 kg/m^2^, blood pressure of 120/70 mmHg, respiratory rate of 20 breaths per minute and a body temperature of 36°C. Her head and neck had no alterations. Cardiopulmonary and abdominal examinations showed normal values. She was overweight, with a hip-to-waist ratio of >0.9. She had no clinical manifestations of peritoneal damage, palpable mass, hepato or splenomegaly. Her genitalia had no alterations and her musculoskeletal system was normal.

She was started on a hypocaloric diet, but her symptoms persisted. We initiated treatment with Spirulina maxima, while continuing her medications for the other diseases. During the initial part of her treatment flatulence was recorded, but no other adverse side-effects were observed.

## Discussion

In all three cases we observed an average decrease in ALT of 41% (Table 1). In our third patient, we observed an initial pathological level of ALT with a final decrease of 34%. Concerning their lipid profiles, we observed a decrease in TAG, TC, LDL-C and TC and/or HDL-C ratios, with an average of 19%, 16%, 22% and 18%, respectively.

**Table 1:** Initial and final plasma values seen in patients

	Case 1			Case 2			Case 3		
**Parameters**	**Initial**	**Final**	**Δ%**	**Initial**	**Final**	**Δ%**	**Initial**	**Final**	**Δ%**

ALT (U/L)	46.3	21.5	-54	35.6	22.6	-37	133.3	88.0	-34
TAG (mg/dL)	76.9	52.9	-32	171.0	146.4	-15	129.8	114.3	-12
TC (mg/dL)	216.3	209.0	-4	275.6	205.6	-26	250.6	200.9	-20
HDL-C (mg/dL)	48.1	55.6	+15	38.6	36.3	-6	49.1	47.6	-3
TC/HDL-C	4.5	3.8	-16.5	7.1	5.7	-20.7	5.1	4.2	-17.3
LDL-C (mg/dL)	152.8	142.8	-7	203.0	136.3	-33	174.9	130.1	-26

ALT: alanine aminotransferase, TAG: triacylglycerols, TC: total cholesterol, HDL-C: cholesterol associated to high-density lipoprotein, LDL-C: cholesterol associated to low-density lipoprotein. Δ%: percentage of change at each parameter.

Our first patient's HDL-C increased by 15% between its initial and final levels. For patients 2 and 3, however, we observed a decrease in their final HDL-C levels. Nevertheless, all patients showed a decrease in the TC-to-HDL-C ratio, as already mentioned above.

**Figure 1:**
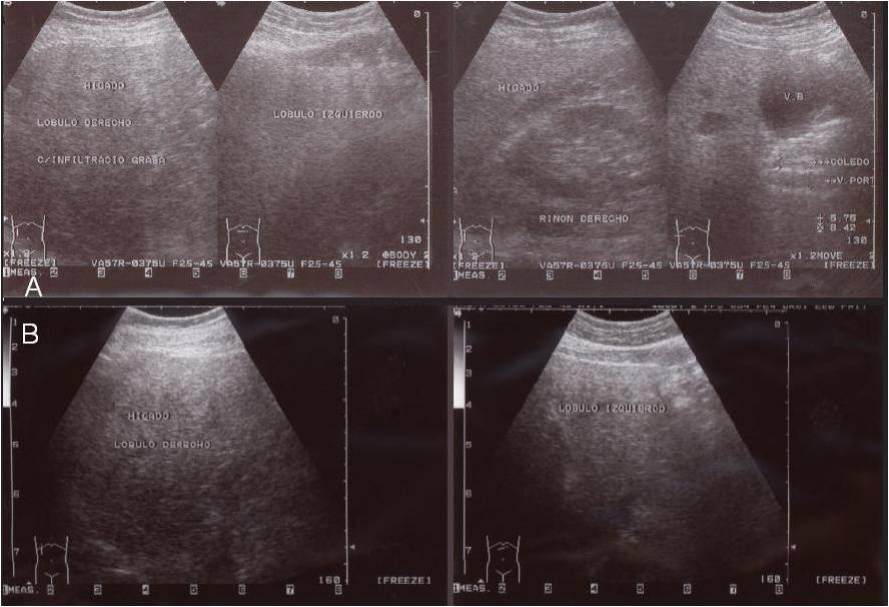
**(A) Patient with non-alcoholic fatty liver disease detected by abdominal ultrasonography**. An increased hepatic parenchymal echotexture by fat infiltration could be appreciated ("brilliant liver"). (B) The same patient after treatment with Spirulina maxima. Notice the attenuation of the previous sonographic pattern.

**Figure 2:**
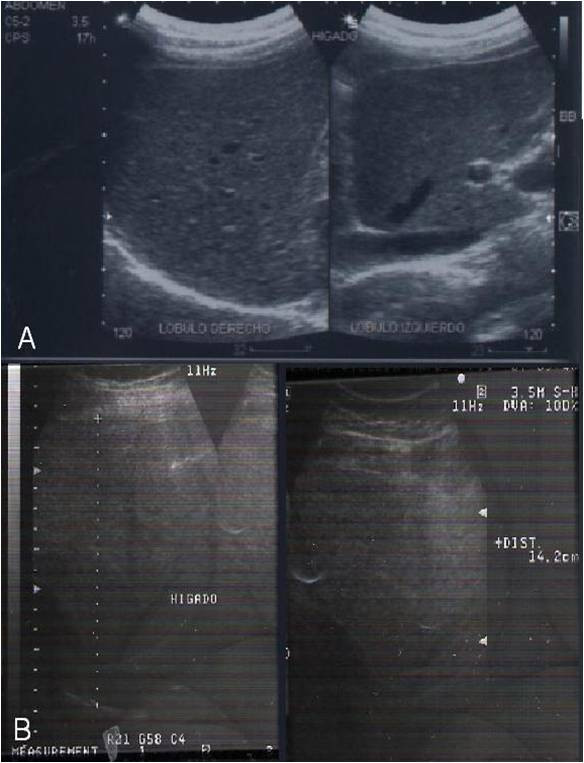
**(A) Abdominal ultrasonography of a patient with non-alcoholic fatty liver disease**. (B) After a daily administration of Spirulina maxima, a normal liver parenchymal texture could be observed on an ultrasonography.

All patients demonstrated a reduction of the symptoms that were indicative of NAFLD (Figures 1 and 2). In two of them, a reduction in parenchyma heterogeneity could be observed, while in the third patient a complete resolution of the "brilliant liver" was recorded. We decided not to take liver biopsies because of the high risk it represents in ambulatory patients and the lack of technical and medical equipment in our laboratory to control possible complications. Beneficial results on plasma indicators and changes observed on liver ultrasonography are in agreement with the hepatoprotective effect of Spirulina demonstrated previously [11] and suggest its potential therapeutic use.

Furthermore, other beneficial effects of Spirulina, such as its antihyperlipidemic and antihypertensive effects, were demonstrated at all doses [12].

Although the exact biochemical mechanism by which Spirulina reduces lipid levels is not well understood, some studies have presumed that its high C-phycocyanin content inhibits pancreatic lipase activity [13]. Its C-phycocyanin content is also presumed to act together with glycolipid hemoglobin (Hb)-2, leading to a decrease in jejunal cholesterol absorption and ileal bile acid reabsorption [14]. These could partly explain changes seen in the ultrasonographies, because of the attenuation of fatty acid flux to the liver, with subsequent decreases in lipid storage. However, this hypothesis does not explain all the physiopathological changes observed among patients with NAFLD.

In another issue, oxidative stress plays a fundamental role in the pathogenesis of fatty liver as observed in patients with NAFLD who have increased levels of malonic dialdehyde, 4-hydroxynonenal, tyrosine nitrated proteins and hydroxydeoxyguanosine. Premkumar *et al*. found that Spirulina fusiformis protects against chemical-induced genotoxicity in mice by increasing the activity of cellular antioxidant enzymes like superoxide dismutase, catalase and glutathione peroxidase [15]. In our laboratory, similar changes in several tissues are being observed on these enzymes resulting from the use of Spirulina. These could be the basis for the hepatoprotective effects induced by Spirulina due to the attenuation of the so-called "second hit" induced by ROS. At this point it is thought that Spirulina could act as a possible supplement in NAFLD treatment. However, studies to evaluate this and other hypotheses are currently still being performed in our laboratory.

## Conclusion

Spirulina maxima showed a therapeutic effect in patients with NAFLD as evidenced by ultrasonography and aminotransferase data. It also showed hypolipidemic effects in the patients described in this case report. To the best of our knowledge, Spirulina maxima could be considered as an alternative treatment for patients with NAFLD and dyslipidemic disorders.

## Consent

Written informed consent was obtained from our patients for publication of this case report and any accompanying images. Copies of the written consent are available for review by the Editor-in-Chief of this journal.

## Competing interests

The authors declare that they have no competing interests.

## Authors' contributions

AFH participated in the collection and analysis of data and in writing the manuscript. PTD participated in the collection, design, analysis and interpretation of data and also in writing the manuscript. MJO participated in the design, analysis and interpretation of data and in writing the manuscript. All authors read and approved the final manuscript.
